# Textural, Color and Sensory Features of Spelt Wholegrain Snack Enriched with Betaine

**DOI:** 10.3390/foods11030475

**Published:** 2022-02-06

**Authors:** Jovana Kojić, Miona Belović, Jelena Krulj, Lato Pezo, Nemanja Teslić, Predrag Kojić, Lidija Peić Tukuljac, Vanja Šeregelj, Nebojša Ilić

**Affiliations:** 1 Institute of Food Technology, University of Novi Sad, Bulevar Cara Lazara 1, 21000 Novi Sad, Serbia; miona.belovic@fins.uns.ac.rs (M.B.); jelena.krulj@fins.uns.ac.rs (J.K.); nemanja.teslic@fins.uns.ac.rs (N.T.); lidija.peictukuljac@fins.uns.ac.rs (L.P.T.); nebojsa.ilic@fins.uns.ac.rs (N.I.); 2 Institute of General and Physical Chemistry, University of Belgrade, Studentski Trg 12-16, 11000 Beograd, Serbia; latopezo@yahoo.co.uk; 3 Faculty of Technology, University of Novi Sad, Bulevar Cara Lazara 1, 21000 Novi Sad, Serbia; kojicpredrag@uns.ac.rs (P.K.); vanjaseregelj@tf.uns.ac.rs (V.Š.)

**Keywords:** extrusion, snack, betaine, functional foods

## Abstract

The influence of different extrusion parameters, including screw speed (250–750 rpm), feed rate (15–25 kg/h) and feed moisture content (15–25%), on the textural and color properties of spelt wholegrain snack products produced on a co-rotating twin-screw extruder with added betaine was investigated. In order to determine the relative influence of input variables in the artificial neural network (ANN) model, Yoon’s interpretation method was used, and it was concluded that feed moisture content has the greatest influence on L* values, while screw speed has the greatest influence on a* and b* values. The softest samples were obtained at the lowest moisture content. Sensory analysis was carried out on selected samples, and it showed that betaine addition did not intensify the bitter taste. The sample with the largest expansion exhibited the lowest hardness and chewiness before and after immersion in milk, and this sample is the most suitable for enrichment with betaine.

## 1. Introduction

Recently, there has been an increasing amount of interest in the replacement of extruded products based on corn grits, which are the most common ones in the market, with nutritional, rich, cereals-based extrudates. Numerous cereals (such as wheat, amaranth and quinoa) have been used to improve the nutritional value and textural properties of extruded snack without reducing product quality in terms of organoleptic properties and consumers acceptability [1,2,3,4]. Thus far, the influence of spelt flour addition on the physical and rheological properties of extruded products based on corn grits has been investigated [5]. In comparison with common wheat, spelt flour has a higher content of protein (especially prolamin) and some amino acids (proline, glutamic acid, tyrosine and aspartic acid), as well as vitamin B, fiber, lipids and mineral elements, and it also has higher bioavailability [6,7,8,9]. The high content of nutritionally valuable components makes spelt flour suitable for the production of a wide range of food products. Although described as poorer in technological quality compared to common wheat, spelt flour is used for the production of pasta, bread, snacks and other food products [8,10,11]. In the last few years, cultivation of spelt flour has increased in Serbia, and a large number of studies have been conducted related to the protective role of the spelt husk of grain [12], for its use in bakeries [13], pasta production [14,15], as well as in solving byproduct issues by pelleting spelt grain husks [16]. Foods based on cereals have been presented as the largest source of betaine in the Western diet [17].

Betaine as a bioactive compound provides many health benefits. The main role of betaine in the human organism is to supply methyl groups for essential physiological processes [18]. The requirements of the organism cannot be satisfied with the endogenous synthesis of betaine, and therefore, its intake is necessary through diet. In the 2017 study of Kojić et al., the following order was determined among cereals in terms of the highest betaine content, with spelt flour at the top: buckwheat < millet < wheat < oats < rye < barley < amaranth < spelt [19]. Functional snack products from spelt wholegrain flour with the addition of betaine have been produced to satisfy the need for the recommended daily intake of betaine of 1500 mg in accordance with Commission Regulation (EU) No 432/2012. In previous work, it was shown that enriched spelt-flour-based extrudates satisfy the recommended daily intake of betaine [20]. Many critical parameters during extrusion, such as the feed rate of the mixture, screw speed and temperature in the barrel and die, affect the sensory properties of extrudate products which are primarily related to taste, texture and color.

Qualitative evaluation of snack products includes sensory, instrumental and microstructural characterization, which represent the final evaluation to determine consumer acceptability. In order to obtain a sensory profile of snack product samples, an objective sensory evaluation needs to be performed using a panel of trained evaluators. Human perception of a product is often closely related to the instrumental analysis of texture. Instrumental texture determination is an objective, fast and relatively inexpensive analysis of the characteristics of final products [21]. Color is one of the most important attributes of food products, providing information regarding the degree of cooking of the product and appearance and freshness of food [22]. It is a very important quantitative characteristic of extrudate quality that is directly related to consumer acceptance [23]. In addition, a change in food color may be a qualitative indicator of the extent of deterioration in food quality due to heat treatment [24]. Since human perception of color is subjective and individual, instrumental techniques for defining the color of a product provide more reliable results. The effects of extrusion parameters and the application of different raw materials on the color of extruded products have been the subject of numerous studies [25,26,27,28,29,30]. The main aim of this research was to evaluate the influence of extrusion cooking parameters (moisture, feed rate and screw speed) on the texture, color and sensory characteristics of snack products based on wholegrain spelt flour with added betaine.

## 2. Materials and Methods

### 2.1. Extrusion Processing—Experimental Design

Spelt flour enriched with betaine (9% *w*/*w* addition) was extruded using a co-rotating twin-screw extruder (Bühler BTSK 30/28D, 7 sections, length/diameter ratio = 28:1, Bühler, Uzwil, Switzerland). The extruder contains two temperature control units (the first unit-controlled temperature in sections set at 60 °C and the second set temperature at 120 °C). Screw configuration specially designed for the production of directly expanded snack products was used (the die opening diameter was 4 mm). A betaine addition of 9% was chosen in accordance with our preliminary trials with wholegrain spelt flour enriched with 1% of betaine, taking into account betaine loss during the extrusion cooking process. Before the extrusion process, the blends were mixed in a twin-shaft paddle mixer that is part of the laboratory vacuum coater (model F-6-RVC, Forberg International AS, Oslo, Norway). Total creation of snack products from spelt wholegrain flour with added 9% *w*/*w* betaine that can be beneficial to the human health and contribute to the recommended daily betaine intake was successfully carried out [23]. The content of betaine was measured by the developed and validated HPLC-ELSD method, and it was in the range from 1248.0 to 1543.1 mg/40 g [19]. 

The effects of the three extrusion factors, i.e., moisture (M; 15–25%), feed rate (FR; 15–25 kg/h) and screw speed (SS; 250–750 rpm), on hardness and color coordinates L*, a* and b* during the extrusion process of snacks was studied. The experimental data used for the analysis were fully determined using a central composite rotatable design (CCRD; α = 1.682), explained with eight cube points, six axial points and three central points (Table 1). The CCRD experimental design was applied to limit the number of samples to a value of 17 that was sufficient for the calculation of the second-order polynomial coefficients in the model and to develop the artificial neural network (ANN). The RSM model describes the effects of process variables on the observed responses, determines interrelationships between process variables and represents the combined effect of all process variables to responses. The developed ANN consisted of three layers (input, hidden and output) with hyperbolic tangent function as the activation function. The Broyden–Fletcher–Gol–dfarb–Shanno (BFGS) calculation showed better model criteria than other training algorithms, such as Levenberg–Marquardt, Bayesian regularization, etc. Having in mind that the ANN results, including weight values, depend on the initial assumptions of parameters and number of hidden neurons, each topology was run several times to avoid overfitting. The coefficient of determination was higher than 0.9 for all ANN runs. In the extrusion process of input-toward-outputs, the ANN was implemented in Yoon’s interpretation method to determine the relative influence of input process variables. The following equation was used:

$$RI_{ij}=\frac{\sum_{k=0}^{n}\left(w_{ik}w_{kj}\right)}{\sum_{i=0}^{m}abs\sum_{k=0}^{n}\left(w_{ik}w_{kj}\right)}100\%$$

where *RI_ij_* is the relative importance of the *i*th input variable on the *j*th output, *w_ik_* is the weight between the *i*th input and the *k*th hidden neuron and *w_kj_* is the weight between the *k*th hidden neuron and the *j*th output [31].

### 2.2. Characterization of Extrudates

#### 2.2.1. Textural Properties

Snack hardness was determined by diametric compression on a TA-XT.2, Texture Analyzer (Stable Micro Systems, Godalming, Surrey, UK) in accordance with the method described by Svihus et al. (2004) [32]. The hardness of the whole snack product (13.5–15.8 mm height, 7.08–7.72 mm diameter) was determined in accordance with a modified method (dry catfood_CTF1_P35). In all, 15 extrudates were taken from each sample, and 3 individual extrudates were placed horizontally on a flat surface of the device and then compressed with a cylindrical probe made of stainless steel with a diameter of 45 mm, load cell of 50 kg and trigger force of 100 g. The hardness of the sample is expressed as the mean value of 15 measurements and is expressed in kilograms. The parameters of the instrument adjustment during the test were as follows: pre-test speed: 2.0 mm/s; test speed: 1 mm/s; post-test speed: 10 mm/s, probe path: 2.5 mm.

#### 2.2.2. Color Measurement

The color of wholegrain spelt flour with the addition of betaine (9% *w*/*w*) and grounded snack products was determined in ten replicates using Chroma Meter CR-400 (Konica Minolta Co., Ltd., Osaka, Japan) and a suitable extension (CR-A50), adapted for measurements of this type of sample in the CIE L* a* b* color space. Total color change (ΔE) between flour and betaine blend and spelt wholegrain flour was calculated based on the following formula:

ΔE = [(L − L_0_)^2^ + (a − a_0_) + (b − b_0_)^2^]^1/2^ (1)

where subscript zero indicates the color parameters of the raw material blend.

#### 2.2.3. Sensory Evaluation of Snack Products Using a Panel of Trained Evaluators

Eight trained panelists, between 25 and 50 years old, from the Institute of Food Technology in Novi Sad, participated in the examination of the sensory properties of snack products. The panelists had more than 4 years of experience in working with commercial products and products developed within scientific research projects. Their training included exercises in identifying, developing terminology and evaluating the intensity of sensory attributes. The panelists had additional training on snack products for the purposes of this study. Two-hour sessions were held to establish the sensory terminology for the tested snack products. Initially, panelists used descriptors from previously published papers with similar topics [33,34] as a starting point, and they could keep, delete or add any descriptor.

A consensus approach was used to determine the final descriptors for snack products. The panel leader led a discussion of each descriptor in order to determine the appropriateness of the terms, definitions and assessment techniques. A final list of descriptors with definitions is given in Supplementary Table S1, which were used by the panel to evaluate all samples in terms of intensity. Intensity assessment was performed using an unstructured linear scale with points 0—imperceptible and 100—very intense. Since it is predicted that the created snack products will be consumed after immersion in milk, the sensory evaluation consisted of two parts.

In the first part, 6 selected attributes of snack products before immersion in milk were evaluated (color, hardness, chewiness, sweet taste, bitter taste). In the second part, 5 g of flips product was immersed in 50 mL of milk (1.5% milk fat) at room temperature. After 5 min, the attributes describing taste and texture were re-evaluated (hardness, chewiness, sweet taste, bitter taste). Distilled water was used to clean the mouth between samples during the evaluation. The assessment was performed in a sensory testing laboratory with appropriate control of environmental conditions [35].

### 2.3. Statistical Analysis

Statistical analysis was obtained by analysis of variance (ANOVA) followed by Tukey’s Test. The results, expressed as mean ± standard deviation, were considered statistically significant with *p* ≤ 0.05. Different letters indicate significant differences in the results (*p* ≤ 0.05).

In order to obtain a better insight into the relationship between sensory properties, instrumentally measured quality parameters (diameter, color and textural properties) and betaine content in snack products, principal component analysis (PCA) was performed using the PanelCheck software (version 1.4.0, Nofima Mat, Norway, Norway, 2010, https://www.panelcheck.com/, accessed on 27 December 2021).

## 3. Results and Discussion

### 3.1. Impact of Process Conditions on Texture Properties of Extrudates

Texture is an important sensory indicator for the quality of snack products. In snack products, expansion is desirable, and texture plays an important role in terms of consumer acceptance [36]. The most commonly used tests to measure the texture of snack are cutting or shear tests, compression and puncture tests. There is no single term that describes the texture of extruded snack products, and the most common terms are hardness, brittleness and crunchiness [34]. In this study, the hardness of snack products from wholegrain spelt flour with the addition of betaine was determined through diametrical compression. Table 1 presents the experimental hardness values for the obtained snack products.

**Figure 1:**
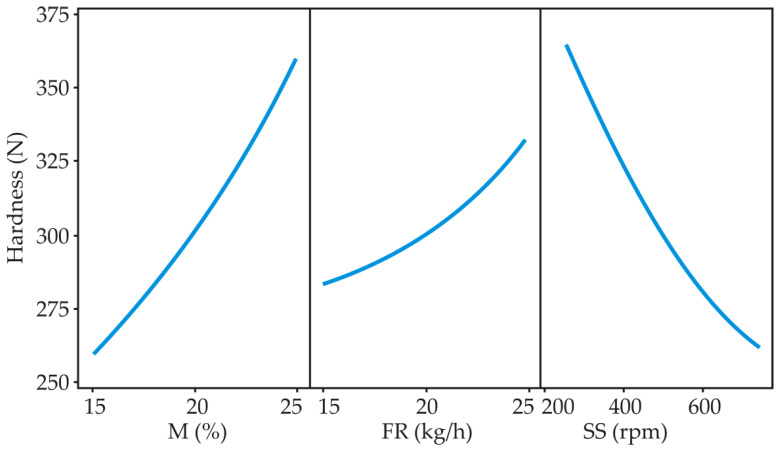
Influence of process parameters moisture (M), feed rate (FR) and screw speed (SS) on hardness.

Figure 1 shows the influence of process parameters (M, FR and SS) on the expansion index, bulk density and hardness of the snack products. A tendency of increasing hardness with increasing feed moisture content (M, %) can be observed. This result is in agreement with the results for bulk density and the expansion index, which has been previously published in our snack from wholegrain spelt flour with the addition of 9% of betaine (Table 1) [37,38]. Namely, a smaller expansion index occurs with an increase in moisture content, and an increase in bulk density with a decrease in the expansion index, which is confirmed in our study. A negative correlation between expansion index and bulk density was observed in our study (r = −0.785; *p* = 0.000 (*p* < 0.001)). Liu et al. (2011) also link the results for bulk density and the expansion index with hardness, which is confirmed in our results through positive correlation between hardness and bulk density (=+0.736; *p* = 0.001 (*p* < 0.01)) (Supplementary Figure S1) [39].

Numerous studies have confirmed that the hardness of the extrudate increased with increasing feed moisture content [40,41]. Increasing the feed moisture content leads to plasticization of the sample, forming a protective layer and compressing the sample, resulting in a high density and hardness of rice [42] and wheat extrudates [2]. The results showed that low hardness was associated with low feed moisture and high screw speed (Figure 1). As screw speed increases, viscosity decreases, which results in lower density and less hardness of extrudate. By contrast, with an increase in feed rate, viscosity increases, giving extrudates with high density and hardness. As screw speed increases, the sample expands and thus becomes softer, while with an increase in feed rate, the barrel of the extruder is filled, and therefore, pressure increases, which leads to the material being compressed and firm. Ding et al. (2006) concluded that feed rate and screw speed have a significant effect on the hardness of wheat extrudate [2]. Diaz et.al (2013) showed that changes in the hardness of extrudates containing kañiwa were caused by screw speed more than changes in feed moisture content [4].

An analysis of operating parameters on hardness is presented in Table 2. The most influential in the second-order polynomial approximation SOP model for hardness evaluation was the linear term of SS statistically significant at *p* < 0.01 level, as well as the linear term of M (statistically significant at *p* < 0.05 level). The coefficient of determination value (R^2^) for the SOP model was 0.823, which can be considered satisfactory for predicting hardness (Table 2).

**Figure 2:**
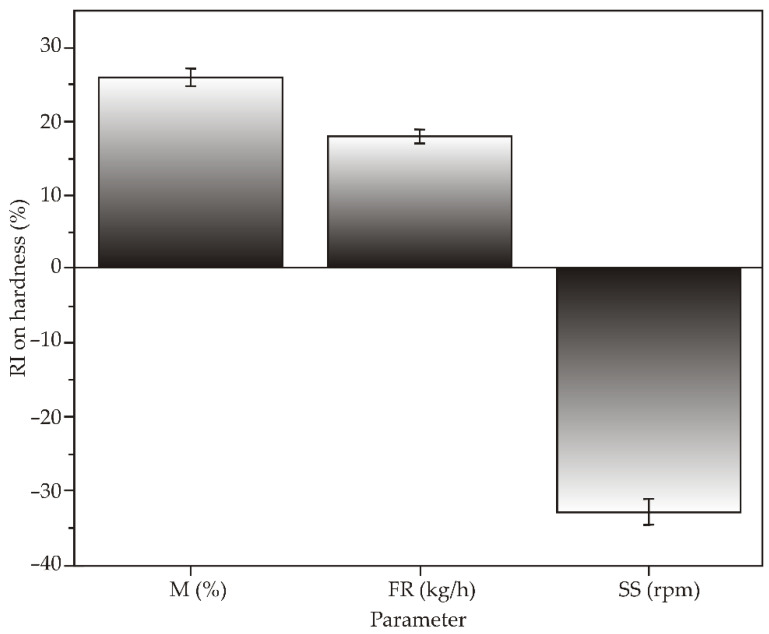
The relative importance of extrusion parameters on hardness using Yoon’s interpretation method.

Moreover, from Figure 2, which presents the relative influence of process parameters on the hardness of extrudates obtained by Yoon’s model, it is clear that feed moisture content and screw speed are the ones that most significantly affect the hardness of the extrudate. In fact, hardness shows a high positive correlation with feed moisture content (increasing with increasing moisture content) and a negative correlation with screw speed (decreasing with increasing screw speed). Feed rate had the smallest effect on the hardness of the extrudate, and the hardness of the extrudate increased with increasing feed rate (Figure 2). These results are in accordance with the results obtained by Brnčić et al. (2006), who concluded that feed moisture content has the greatest positive effect on hardness, while screw speed and temperature have a significant negative effect on hardness [43].

### 3.2. Impact of Process Conditions on Color Properties of Extrudates

The color of extruded products can vary depending on the combination of established parameters such as raw material moisture content, temperature and chemical components of each raw material and their ratio in the mixture. Therefore, it is important to control the color of the ingredients, as well as to monitor the product throughout the production process to obtain and maintain the desired color [44]. The values of lightness (L*) of ground snack products from wholegrain spelt flour with added betaine ranged from 65.04 to 73.50, and the redness value (a*) and yellowness value (b*) of the same samples was in the range of 3.20–4.97 and 15. 71–17.25, respectively (Table 3). The value of L* for the control whole grain flour with 9% betaine was 81.232, while the values of a* and b* were 1.084 and 11.614, respectively. ΔE values calculated between snack products and blend ranged from 10.15 to 17.06, indicating a very pronounced color change. These results are in agreement with the results obtained in the study by Wani and Kumar (2015), who examined the effect of the addition of different vegetable raw materials on the color change of corn, rice and barley extrudates and recorded values of 56.3–71.3 for L*, 4, 44–6.47 for a* and 11.89–19.88 for b* [45].

The presented results indicate that the values of L* after extrusion are reduced, while the values of a* and b* are increased (Table 3). These results are in agreement with the results of Menegassi et al. (2011) and Durge et al. (2013) [44,46]. Changes in the color of extrudates may be related to the potential role of betaine as an amino acid in Maillard reactions and may be due to a reaction between betaine and sugar that contributes to the formation of colored compounds (products of Maillard reactions) that reduce the lightness of extrudates.

**Figure 3:**
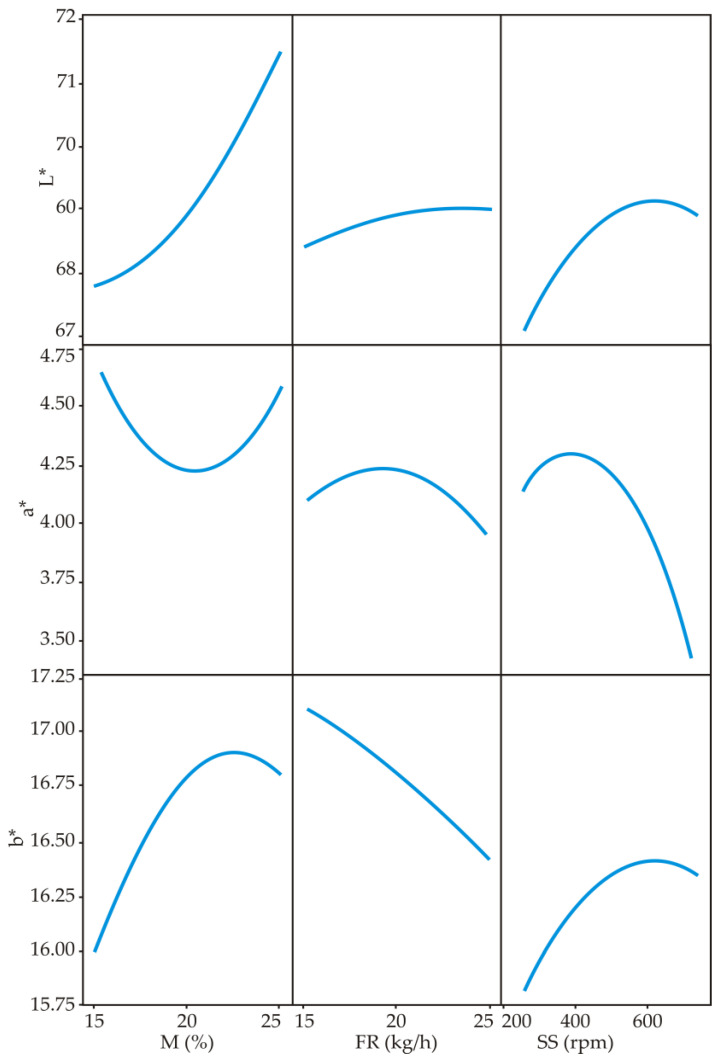
Influence of process parameters moisture (M), feed rate (FR) and screw speed (SS) on L*, a* and b*.

From Figure 3, it is clear that L* and b* color values increased with increasing feed moisture, while parameter a* decreased with increasing feed moisture up to 20% and then started to increase.

Feed moisture is an important factor, and its increase gives a lighter product, i.e., it prevents its darkening and has a protective role in the extrusion process. It is considered that increased feed moisture lowers the temperature of the extrusion process, which in turn reduces the potential for darkening of the product through Maillard reactions between reducing sugars and free amino groups [47].

Increasing screw speed in the extrusion cooking process increased the values of L* and b*, while the value of a* was decreased (Figure 3). As concluded by Gulati et al. (2016), an increasing value of b* with an increase in screw speed may be associated with a lower retention time of the material in the extruder barrel, thus achieving less sample cooking [48]. The obtained results are in accordance with extruded rice flour [49]. Additionally, increasing the feed rate increased the L* values, while a* and b* decreased (Figure 3).

**Figure 4:**
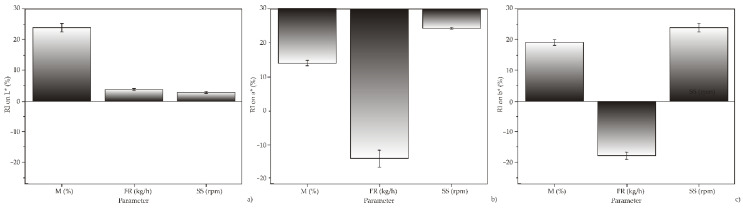
The relative importance of the extrusion parameters on the color parameters (**a**) L*—lightness; (**b**) a*—red/green color; (**c**) b*—yellow/blue color, using Yoon’s interpretation method.

Yoon’s model (Figure 4) has shown that feed moisture content has the greatest influence on the L* values, while screw speed has the greatest influence on the a* and b* values.

Gulati et al. (2016) showed that feed moisture content is the main factor influencing the values of L* and a*, while feed moisture in interaction with temperature was the main factor influencing the value of b* [48].

For L* value evaluation, the most influential was the linear term of M in the SOP model (statistically significant at *p* < 0.1 level), as well as the linear term of SS for the evaluation of value a* (statistically significant at *p* < 0.05 level). For calculating the b* value, the most influential were the linear terms M and SS, as well as the combined effect of these two variables (statistically significant at the level of *p* <0.10), Table 4.

The coefficients of determination for the calculation of L*, a* and b* had values of 0.533, 0.766 and 0.760, respectively, which can be considered relatively satisfactory for predicting the stated color coordinates.

### 3.3. Sensory Evaluation of Snack Products

**Figure 5:**
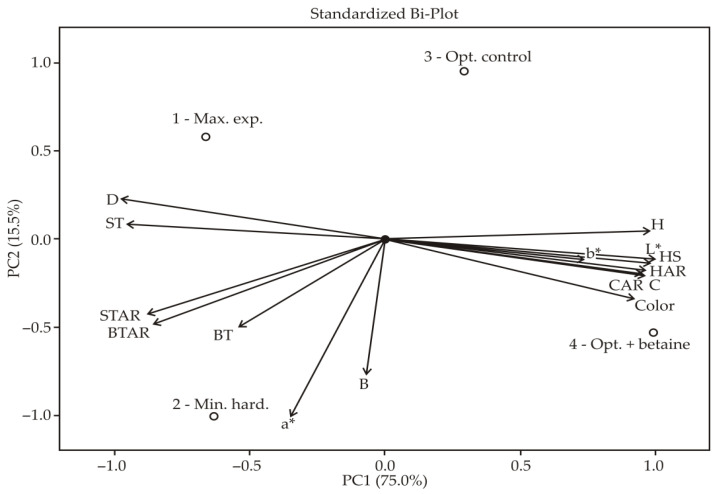
Linear combinations of variables identified via PCA analysis and the position of samples in the factor space (Bi-plot).

The relationship between the diameter, instrumentally measured color and hardness, betaine content and sensory descriptors of the evaluated snack samples was visually presented by linear combinations of variables identified by PCA (Loading Plot) and the position of samples in the factor space (Score plot) together in a Bi-plot (Figure 5). The first two principal components (F1 and F2) explained 90.73% of the total variability, which can be explained by a good selection of sensory variables and a relatively small number of tested samples. If the relationship between variables is considered, three groups can be observed, and it can be concluded that all variables within one group are in a significant positive correlation with each other (r close to +1). The first group of variables consisted of diameter and sweet taste. These two parameters could be related since a higher expansion index occurs when there is a higher degree of starch gelatinization, and starch hydrolysis into fragments with smaller molecular weights and higher sweetness could also occur at the same time [50].

PCA—principal component analysis; B—betaine; BT—bitter taste; BTAR—bitter taste after rehydration; C—chewiness; CAR—chewiness after rehydration; D—diameter; H—hardness; HAR—hardness after rehydration; HS—hardness (sensory); ST—sweet taste; STAR—sweet taste after rehydration.

The second group of variables contained L* and b* color parameters, instrumentally measured hardness and sensory determined color, hardness and chewiness before and after immersion in milk and were opposite to the variables in the first group.

This indicates that the variables in the first and second groups are mutually negatively correlated (r close to −1). Hardness determined by a sensory panel was highly correlated with hardness determined instrumentally, and color intensity perceived by a sensory panel was highly correlated with lightness (L*) and yellow tone intensity (b*), indicating that these instrumental parameters could be used successfully for fast determination of the sensory quality of snack samples. Additionally, this grouping indicated that smaller diameter snack samples (samples 3 and 4) are at the same time harder, tougher (require more time to be masticated) and darker, and that after immersion in milk, the relationship between their mechanical properties remains the same.

The variables in the third group were a* color parameter, betaine content, bitter taste before immersion, as well as bitter and sweet taste after immersion in milk. These correlations suggest that betaine content in snack samples could be related to the perceived bitter taste, and a more pronounced red tone (a* values) could be a consequence of the aforementioned Maillard reactions.

According to the Score plot, the selected sensory descriptors enabled a clear distinction between the obtained snack samples. The sample with the maximum expansion (sample 1) is distinguished, as expected, with the largest diameter, but also with the most pronounced sweet taste. The sample with the lowest hardness (sample 2) is distinguished through bitter taste before and after immersion in milk, as well as through sweet taste after immersion in milk and betaine content. Since this sample had the best rehydration properties (the weakest mechanical properties) and therefore absorbed the largest amounts of milk, both flavors present in the sample came to the fore due to the dissolution of substances that give a sweet and bitter taste in milk.

Snack extruded products that are obtained via an optimized extrusion process (samples 3 and 4) are characterized by higher hardness and chewiness both before and after immersion in milk, as well as by a darker color.

Since sample 1 has the best mechanical properties (largest diameter, the lowest hardness before and after immersion, the lowest chewiness before and after immersion) and a distinctly sweet taste, it can be considered the most suitable for consumers. It is important to note that betaine slightly influenced bitter taste (sample number 4) but reduced the mechanical properties (samples 3 and 4). Additionally, it is assumed that sample 1 would be the most suitable for enrichment with betaine.

## 4. Conclusions

This study confirmed that the operational parameters of the extrusion cooking process (moisture content (M, %), feed rate (FR, (kg/h)) and screw speed (SS, rpm)) affect the success of the sensory experience of the snack product, which is related to texture and color. The softest spelt wholegrain snack was produced at the lowest level of feed moisture content. The results for hardness obtained by Yoon’s model showed that feed moisture content and screw speed are the most influential parameters during the production of spelt wholegrain snacks with added betaine. Decrease in the lightness of the extrudate may be associated with a reaction between betaine and sugar that contributes to the formation of colored compounds. L* color values were decreased after extrusion, while a* and b* values were increased. In addition to satisfying the nutritional recommended daily intake of betaine, it is important to obtain a product that is acceptable in terms of sensory properties. Products obtained via the optimized extrusion process are not rated as the most acceptable, which emphasizes the importance of sensory analysis, which represents the final assessment carried out by consumers. The sample with the largest expansion, lowest hardness before and after immersion and lowest chewiness before and after immersion can be considered the most appropriate for supplementation with betaine and for consumers.

## Figures and Tables

**Table 1:** Experimental EI, BD and hardness values of snack according to the adopted central composite rotatable design (CCRD) experimental plan.

Variables			Product Response			
CCRD Runs	M (%)	FR (kg/h)	SS (o/min)	EI	BD (g/L)	Hardness (N)
1	20	20	500	1.82 ± 0.18 ^a^	328.7 ± 34.5 ^ab^	302.4 ± 21.3 ^bcd^
2	20	20	250	1.54 ± 0.16 ^a^	478.0 ± 51.2 ^c^	372.2 ± 14.9 ^e^
3	17	17	350	1.60 ± 0.17 ^a^	367.0 ± 34.5 ^b^	268.1 ± 16.1 ^ab^
4	20	20	750	1.93 ± 0.18 ^a^	244.4 ± 24.6 ^a^	262.7 ± 20.5 ^ab^
5	20	20	500	1.81 ± 0.20 ^a^	333.1 ± 30.4 ^ab^	302.4 ± 12.8 ^bcd^
6	23	17	350	1.69 ± 0.18 ^a^	359.1 ± 37.2 ^b^	351.4 ± 22.0 ^de^
7	17	17	650	1.90 ± 0.18 ^a^	367.2 ± 38.7 ^b^	300.6 ± 16.8 b^cd^
8	25	20	500	1.72 ± 0.16 ^a^	380.0 ± 40.0 ^bc^	366.9 ± 34.6 ^e^
9	20	25	500	1.78 ± 0.19 ^a^	359.1 ± 34.8 ^b^	342.0 ± 21.3 ^cde^
10	23	17	650	1.88 ± 0.19 ^a^	299.9 ± 28.4 ^ab^	276.7 ± 14.8 ^b^
11	20	15	500	1.83 ± 0.17 ^a^	321.8 ± 31.0 ^ab^	282.8 ± 10.4 ^bc^
12	23	23	650	1.86 ± 0.19 ^a^	298.7 ± 27.3 ^ab^	343.9 ± 15.9 ^de^
13	20	20	500	1.81 ± 0.20 ^a^	341.6 ± 34.9 ^ab^	302.4 ± 27.7 ^bcd^
14	15	20	500	1.92 ± 0.20 ^a^	324.7 ± 29.5 ^ab^	261.8 ± 26.8 ^ab^
15	17	23	650	1.89 ± 0.19 ^a^	249.8 ± 27.1 ^a^	214.3 ± 20.2 ^a^
16	23	23	350	1.62 ± 0.16 ^a^	385.7 ± 35.6 ^bc^	377.0 ± 36.6 ^e^
17	17	23	350	1.82 ± 0.19 ^a^	380.0 ± 41.1 ^bc^	357.1 ± 25.4 ^de^

M (%)—feed moisture, FR (kg/h)—feed rate, SS (rpm)—screw speed, CCRD—central composite rotatable design. Means in the same column with different superscript are statistically different (*p* ≤ 0.05); EI—expansion index, BD—bulk density.

**Table 2:** Analysis of variance for second-order polynomial for hardness calculation.

Terms	df	Hardness
M	1	113.128 *
M^2^	1	1.194
FR	1	28.863
FR^2^	1	0.729
SS	1	122.982 ^+^
SS^2^	1	2.158
M × FR	1	10.557
M × SS	1	0.009
SS × SS	1	23.222
Error	7	64.989
r^2^		0.823

df—degrees of freedom; ^+^ statistically significant at *p* < 0.01 level, * statistically significant at *p* < 0.05 level.

**Table 3:** Experimental color values of snack according to the adopted CCRD experimental plan.

Variable			Product Response				
CCRD Runs	M (%)	FR (kg/h)	SS (o/min)	L*	a*	b*	ΔE
1	20	20	500	69.00 ± 5.65 ^a^	4.18 ± 0.25 ^bc^	16.89 ± 1.35 ^a^	13.68 ± 1.45 ^abcd^
2	20	20	250	66.07 ± 5.20 ^a^	4.01 ± 0.23 ^bc^	15.74 ± 1.30 ^a^	15.98 ± 1.46 ^cd^
3	17	17	350	67.80 ± 4.08 ^a^	4.97 ± 0.30 ^d^	17.00 ± 1.52 ^a^	14.98 ± 1.53 ^bcd^
4	20	20	750	69.10 ± 4.57 ^a^	3.20 ± 0.19 a	16.94 ± 1.27 ^a^	13.42 ± 1.27 ^abc^
5	20	20	500	69.00 ± 5.70 ^a^	4.25 ± 0.26 ^bcd^	16.50 ± 1.02 ^a^	13.55 ± 1.40 ^abcd^
6	23	17	350	69.12 ± 4.46 ^a^	4.25 ± 0.35 ^bcd^	16.41 ± 1.32 ^a^	13.41 ± 1.28 ^abc^
7	17	17	650	68.79 ± 3.97 ^a^	4.30 ± 0.25 ^bcd^	15.76 ± 0.83 ^a^	13.50 ± 1.28 ^abcd^
8	25	20	500	73.50 ± 4.75 ^a^	4.66 ± 0.36 ^cd^	17.14 ± 1.31 ^a^	10.15 ± 1.10 ^a^
9	20	25	500	68.38 ± 5.91 ^a^	3.91 ± 0.22 ^ab^	16.74 ± 1.34 ^a^	14.12 ± 1.28 ^bcd^
10	23	17	650	69.44 ± 3.65 ^a^	3.88 ± 0.30 ^ab^	17.25 ± 1.04 ^a^	13.37 ± 1.39 ^abc^
11	20	15	500	68.28 ± 3.81 ^a^	3.80 ± 0.27 ^ab^	17.07 ± 1.04 ^a^	14.31 ± 1.44 ^bcd^
12	23	23	650	68.75 ± 5.91 ^a^	4.22 ± 0.35 ^bc^	16.53 ± 0.87 ^a^	13.78 ± 1.29 ^bcd^
13	20	20	500	69.00 ± 3.89 ^a^	4.35 ± 0.26 ^bcd^	16.98 ± 1.46 ^a^	13.75 ± 1.50 ^bcd^
14	15	20	500	65.04 ± 5.79 ^a^	4.26 ± 0.21 ^bcd^	15.93 ± 0.98 ^a^	17.06 ± 1.62 ^d^
15	17	23	650	70.38 ± 6.01 ^a^	3.86 ± 0.27 ^ab^	16.31 ± 1.23 ^a^	12.15 ± 1.29 ^ab^
16	23	23	350	69.41 ± 5.47 ^a^	4.23 ± 0.27 ^bc^	15.71 ± 0.99 ^a^	12.90 ± 1.31 ^abc^
17	17	23	350	68.86 ± 5.36 ^a^	4.34 ± 0.26 ^bcd^	15.73 ± 1.09 ^a^	13.44 ± 1.37 ^abc^

M (%)—feed moisture, FR (kg/h)—feed rate, SS (rpm)—screw speed, CCRD—central composite rotatable design. Means in the same column with different superscript are statistically different (*p* ≤ 0.05).

**Table 4:** Analysis of variance for second-order polynomial for color parameter calculation.

Terms	df	L*	a*	b*
M	1	16.576 **	0.004	0.717 **
M^2^	1	0.769	0.225	0.239
FR	1	0.431	0.024	0.534
FR^2^	1	0.060	0.062	0.002
SS	1	3.846	0.612 *	0.664 **
SS^2^	1	1.291	0.300 **	0.521
M × FR	1	1.163	0.242	0.061
M × SS	1	1.015	0.074	0.673 **
FR × SS	1	0.025	0.038	0.405
Error	7	22.756	0.546	1.174
r^2^		0.533	0.766	0.760

df—degrees of freedom; * statistically significant at *p* < 0.05 level; ** statistically significant at *p* < 0.10 level; L*—lightness; a*—red/green color; b*—yellow/blue color.

## Data Availability

The data that support the findings of this study are available from the corresponding author upon reasonable request.

## Supplementary Materials

The following supporting information can be downloaded at: https://www.mdpi.com/article/10.3390/foods11030475/s1; Table S1: Sensory descriptors and definitions used in the sensory analysis of snack product samples; Figure S1: Correlation between (**a**) the expansion index and bulk density and between (**b**) hardness and bulk density.
