# Supplementary material for: Textural, Color and Sensory Features of Spelt Wholegrain Snack Enriched with Betaine
Source: Foods. 2022 Feb 6;11(3):475. doi: 10.3390/foods11030475 (PMC8834531; doi:10.3390/foods11030475)
Supplement: Supplementary file 1 [file foods-11-00475-s001.zip › foods-1558485-supplementary.pdf]

Table S1. Sensory descriptors and definitions used in the sensory analysis of snack product samples.

Descriptor		Definition
Appearance	Color	Color on a scale from light yellow to dark brown
Before immersion in milk		
Texture	Hardness	The force required to crush food using incisor
	Chewiness	The number of chewing needed to swallow food
Taste	Sweet	Basic taste typical of sucrose solution
	Bitter	Basic taste typical of caffeine or quinine solution
After immersion in milk		
Texture	Hardness	The force required to crush food using incisor
	Chewiness	The number of chewing needed to swallow food
Taste	Sweet	Basic taste typical of sucrose solution
	Bitter	Basic taste typical of caffeine or quinine solution

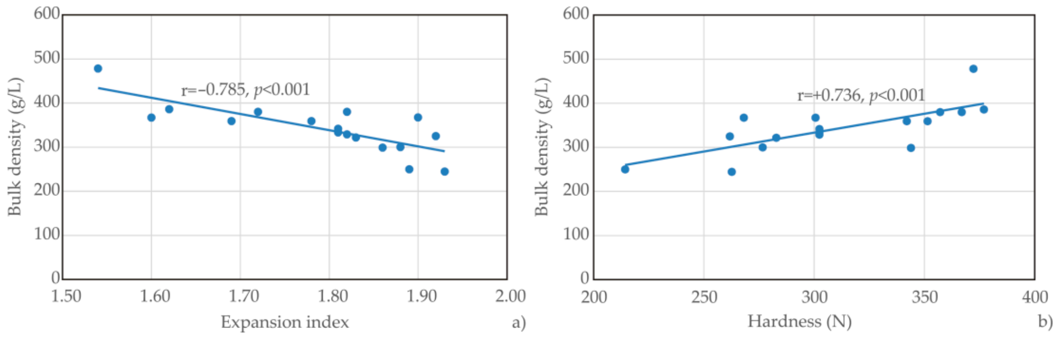

Figure S1. Correlation between (**a**) the expansion index and bulk density and between (**b**) hardness and bulk density.
